# Supplementary material for: Retinal Genomic Fabric Remodeling after Optic Nerve Injury
Source: Genes (Basel). 2021 Mar 11;12(3):403. doi: 10.3390/genes12030403 (PMC7999523; doi:10.3390/genes12030403)
Supplement: Supplementary file 1 [file genes-12-00403-s001.pdf]

Article

Retinal genomic fabrics remodeling after optic nerve injury

Pedro Henrique Victorino[1], Camila Marra[1], Dumitru Andrei Iacobas[2,3], Sanda Iacobas[4], David C. Spray[3], Rafael Linden[1], Daniel Adesse[5,*,†] and Hilda Petrs-Silva[1,*]

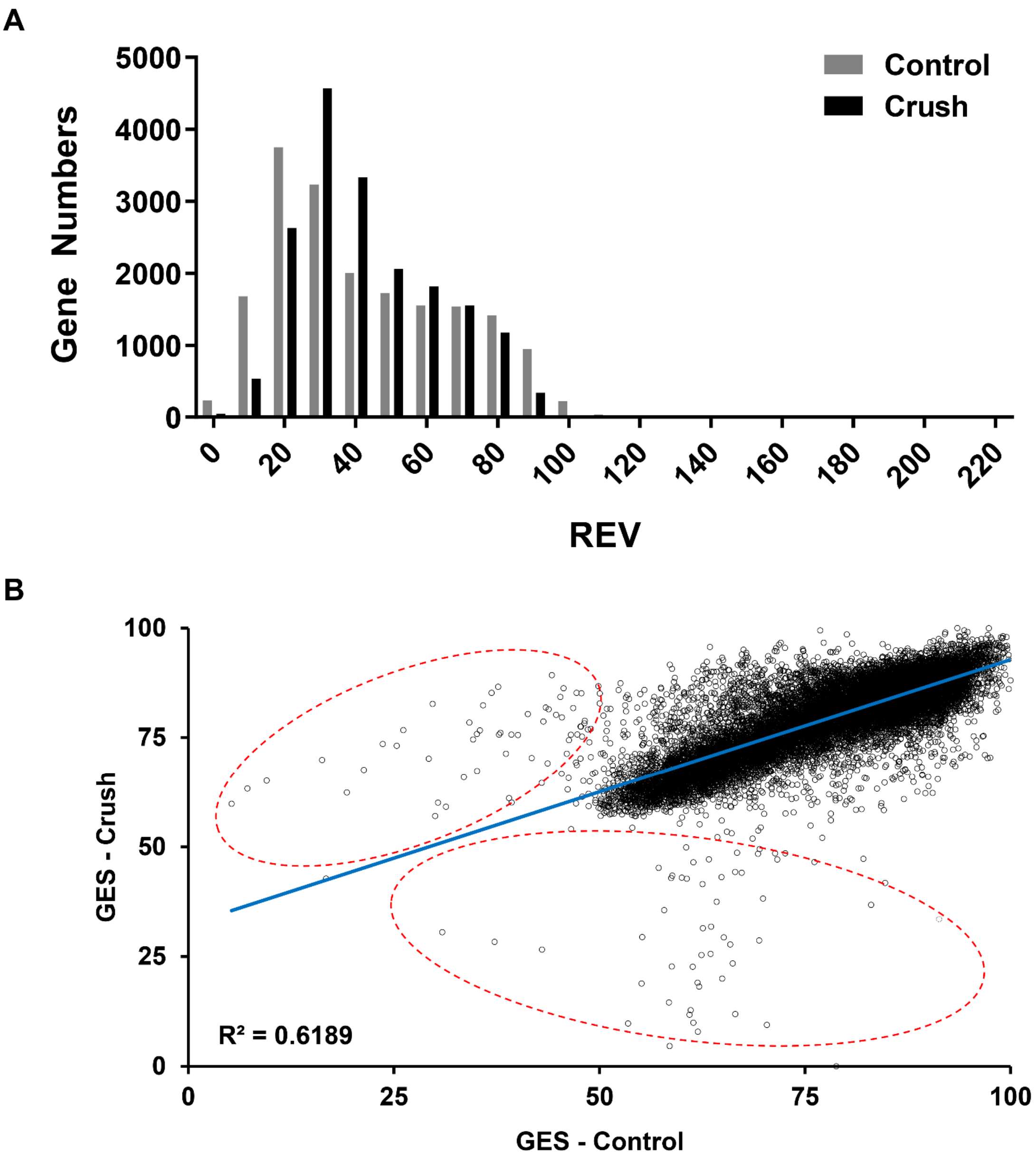

Figure S1: REV and GES of individual genes in each experimental condition. (a) Histogram of the REVs among the four replicas of CTR and ONC experimental groups. REV profile of genes after ONC was very similar compared to that of CTR retinas, with a slight shift towards a higher variability. (b) Linear regression of the GES

of individual genes in each experimental condition indicated a slight decrease of the very stably expressed genes (GES > 95) after ONC. Red circles contain genes that presented the highest change in their expression stability among experimental groups.

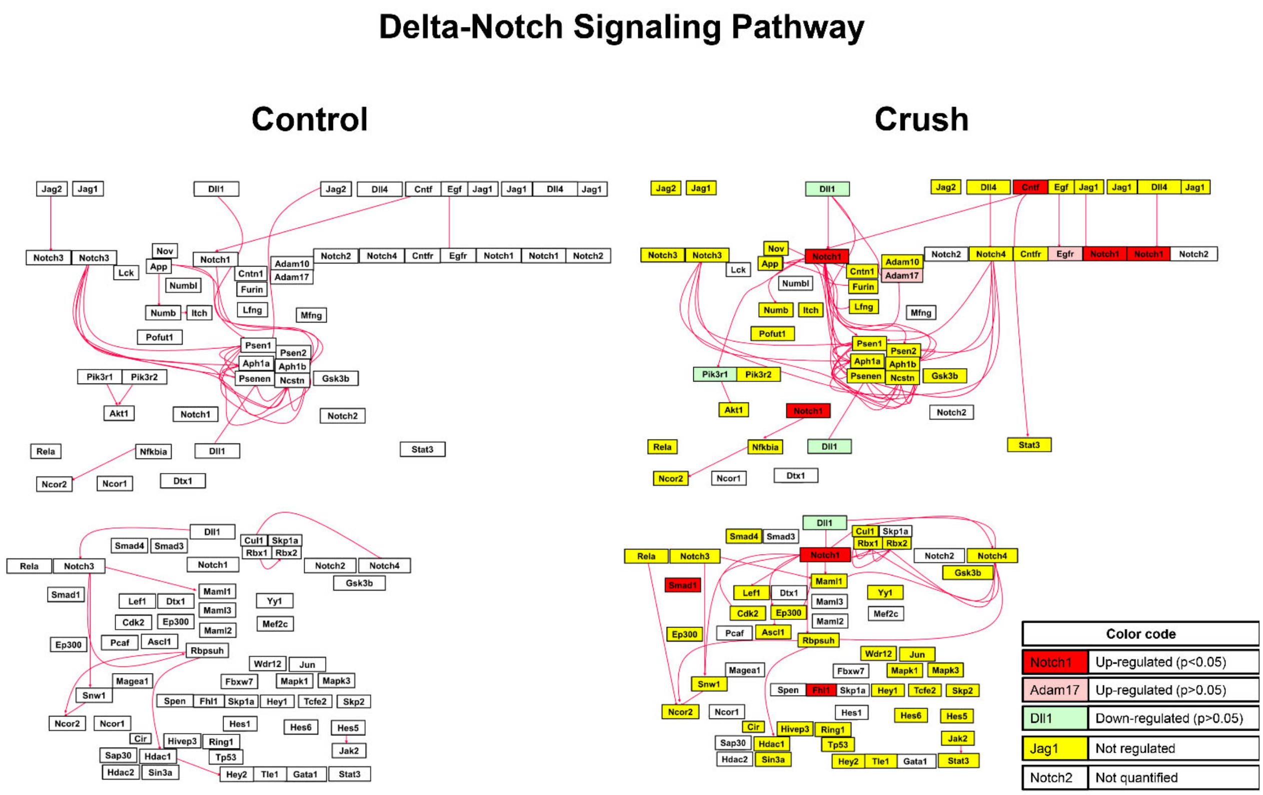

Figure S2. ONC affects the Delta-Notch Signaling Pathway. This pathway was obtained from the WikiPathway platform and used as a template to highlight the effect of the ONC in the retinas. Magenta lanes represent synergistic correlations between pairs of genes. ONC induced an increase in the number of synergistic correlations. Yellow boxes indicate genes that showed no alteration in ONC versus CTR retinas, whereas red-filed boxes represent gene up-regulation (>1.5-fold change, p <0.05).

Oxidative Stress

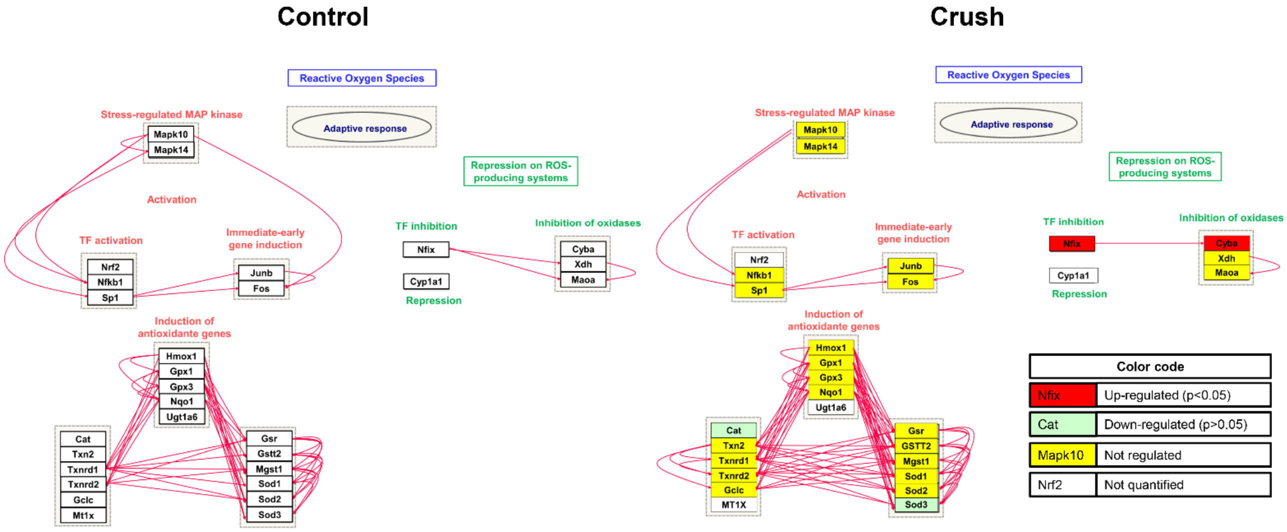

Figure S3. ONC affects the Oxidative Stress. This pathway was obtained from the WikiPathway platform and used as a template to highlight the effect of the ONC in the retinas. Magenta lanes represent synergistic correlations between pairs of genes. ONC induced an increase in the number of synergistic correlations. Yellow boxes indicate genes that showed no alteration in ONC versus CTR retinas, whereas red-filed boxes represent gene up-regulation (>1.5-fold change, p <0.05).

Kit Receptor Signaling Pathway

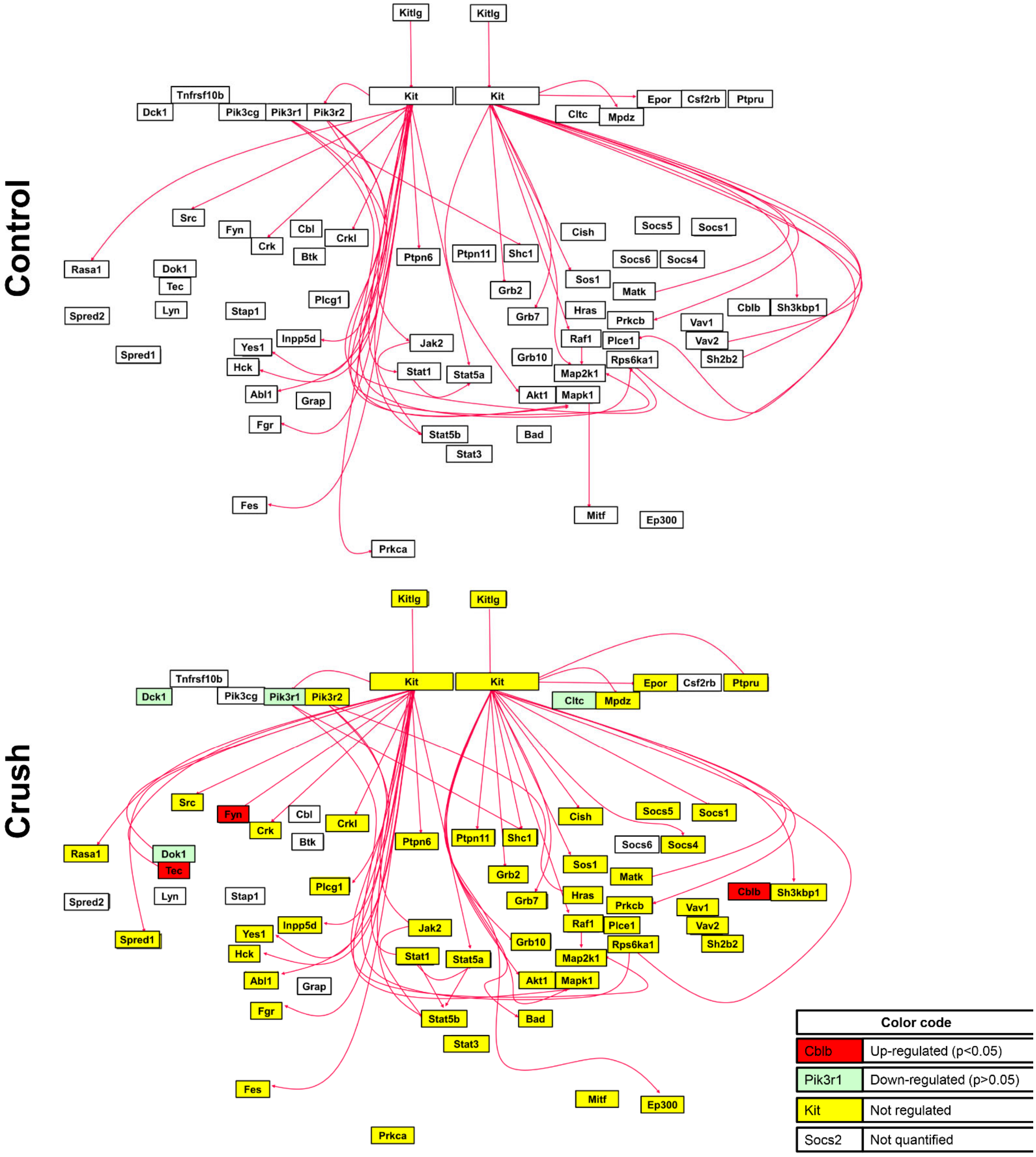

Figure S4. ONC affects the Kit Receptor Signaling Pathway. This pathway was obtained from the WikiPathway platform and used as a template to highlight the effect of the ONC in the retinas. Magenta lanes represent synergistic correlations between pairs of genes. ONC induced an increase in the number of synergistic correlations. Yellow boxes indicate genes that showed no alteration in ONC versus CTR retinas, whereas red-filed boxes represent gene up-regulation (>1.5-fold change, p <0.05).

Table S1. Disease signature of the down-regulated genes

Term	Overlap	p value
Glaucoma associated with systemic syndromes DOID-1686 mouse GSE26299 sample 491	38/341	1.75^{-50}
Glaucoma associated with systemic syndromes DOID-1686 mouse GSE26299 sample 490	23/268	6.73^{-27}
Glaucoma associated with systemic syndromes DOID-1686 mouse GSE26299 sample 488	25/328	5.36^{-28}
Spinal Cord Injury C0037929 rat GSE2599 sample 426	18/351	5.15^{-17}
Neurological pain disorder C0423704 rat GSE2636 sample 129	17/338	5.95^{-16}

Table S2. Disease signature of the up-regulated genes

Term	Overlap	p value
Glaucoma associated with systemic syndromes DOID-1686 mouse GSE26299 sample 490	23/332	1.28^{-17}
Neurological pain disorder C0423704 rat GSE18803 sample 212	28/411	3.79^{-21}
Glaucoma associated with systemic syndromes DOID-1686 mouse GSE26299 sample 489	21/350	6.67^{-15}
Diabetic Retinopathy C0011884 rat GSE1979 sample 189	23/346	3.19^{-17}
Glaucoma associated with systemic syndromes DOID-1686 mouse GSE26299 sample 491	20/259	2.48^{-16}
